# Diversity in clinical manifestations of autoinflammatory syndromes

**DOI:** 10.1186/1546-0096-9-S1-P306

**Published:** 2011-09-14

**Authors:** S Boiu, B Neven, S Compeyrot-Lacassagne, R Mouy, B Bader-Meunier, P Quartier

**Affiliations:** 1 Department of Immunology, Hematology and Rheumatology, Necker-Enfants Malades Hospital, Paris, France

## Background

The autoinflammatory syndromes (AIS) include monogenic and polygenic disorders characterized by primary dysfunction of the innate immune system.

## Aim

To describe the spectrum of AIS in a Pediatric Rheumatology reference center.

## Methods

Medical records of AIS patients followed until November 2010 were studied.

## Results

Fifty six patients were included: 17 CAPS, 4 TRAPS, 5 HIDS, 18 FMF, 6 CRMO, 2 SAPHO and 4 Behcet. The median follow-up period was 2 years (0-14 years). The male/female ratio was 20/36. The median age was 2.5 years at disease onset and 4 years at diagnosis. Family history was positive in 34% of patients. Clinical manifestations included fever (79%), mucocutaneous (61%), musculoskeletal (77%), ocular (34%), cardiorespiratory (12%), gastrointestinal (62%), neurological (41%) and genitourinary (2%) findings, lymphadenopathy with/or hepatosplenomegaly (16%) and growth impairment (27%). Seven patients presented severe manifestations: neonatal peritonitis (1 CAPS), pancreatitis (1 TRAPS), acute glomerulonephritis (1 FMF), complicated Henoch-Schönlein purpura (1 FMF), peritoneal adhesions with intestinal occlusion (1 FMF), periorbital pain (1 CRMO), and cerebral thrombosis (1 Behcet). One mutation was found in 93% of CAPS, and in all TRAPS patients. Two mutations were present in 11% of FMF, and in all HIDS patients. 43% of patients received colchicine, 23% steroids, and 54% biologics (Anakinra, Canakinumab, Etanercept). 57% of patients were in complete and 41% in partial remission.

## Conclusion

AIS in children are associated with a broad spectrum of symptoms. A detailed history, thorough review of clinical symptoms and molecular testing of specific causative genes can provide early and accurate diagnosis.

